# Classification of comorbidity in obsessive–compulsive disorder: A latent class analysis

**DOI:** 10.1002/brb3.1641

**Published:** 2020-05-13

**Authors:** Lucas J. B. van Oudheusden, Rens van de Schoot, Adriaan Hoogendoorn, Patricia van Oppen, Maarten Kaarsemaker, Gerben Meynen, Anton J. L. M. van Balkom

**Affiliations:** ^1^ Department of Psychiatry, Amsterdam Public Health Amsterdam UMC, Vrije Universiteit Amsterdam Amsterdam The Netherlands; ^2^ GGZ inGeest Amsterdam The Netherlands; ^3^ Department of Methods and Statistics Utrecht University Utrecht The Netherlands; ^4^ Vincent van Gogh Institute Mental Health Care Centre Noord‐ en Midden‐Limburg Venray The Netherlands; ^5^ Faculty of Humanities VU University Amsterdam Amsterdam The Netherlands; ^6^ Willem Pompe Institute for Criminal Law and Criminology Utrecht University The Netherlands

**Keywords:** classification, comorbidity, latent class analysis, obsessive–compulsive disorder

## Abstract

**Objective:**

Patients with OCD differ markedly from one another in both number and kind of comorbid disorders. In this study, we set out to identify and characterize homogeneous subgroups of OCD patients based on their comorbidity profile.

**Methods:**

In a cohort of 419 adult subjects with OCD, the lifetime presence of fifteen comorbid disorders was assessed. Latent class analysis was used to identify comorbidity‐based subgroups. Groups were compared with regard to core clinical characteristics: familiality, childhood trauma, age at onset, illness severity, OCD symptom dimensions, personality characteristics, and course of illness.

**Results:**

The study sample could be divided in a large group (*n* = 311) with a low amount of comorbidity that could be further subdivided into two subgroups: OCD simplex (*n* = 147) and OCD with lifetime major depressive disorder (*n* = 186), and a group (*n* = 108) with a high amount of comorbidity that could be further subdivided into a general anxiety‐related subgroup (*n* = 49), an autism/social phobia‐related subgroup (*n* = 27), and a psychosis/bipolar‐related subgroup (*n* = 10). Membership of the high‐comorbid subgroup was associated with higher scores on childhood trauma, illness severity, and the aggression/checking symptom dimension and lower scores on several personality characteristics.

**Conclusion:**

Grouping OCD patients based on their comorbidity profile might provide more homogeneous, and therefore, more suitable categories for future studies aimed at unraveling the etiological mechanisms underlying this debilitating disorder.

## Graphical abstract

In this study, we set out to identify and characterize homogeneous subgroups of OCD patients based on their comorbidity profile. This might provide more homogeneous and therefore more suitable categories for future studies aimed at unraveling the etiological mechanisms underlying this debilitating disorder.

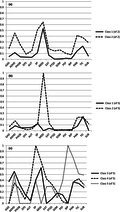

## SIGNIFICANT OUTCOMES

- Based on comorbidity profiles, five distinct subgroups of OCD patients could be distinguished.
- Group membership was associated with differences in core clinical characteristics and course of illness.

## LIMITATIONS

- Subjects with a comorbid psychotic and/or bipolar disorder might be underrepresented in our sample.
- The dataset lacked information on the presence of several other comorbid diagnoses of interest, most notably body dysmorphic disorder, grooming disorders, illness anxiety disorder, and impulse‐control disorders.

## 1 INTRODUCTION

Obsessive–compulsive disorder (OCD) is, in many respects, a heterogeneous mental disorder. Reducing clinical heterogeneity might help improve the understanding of etiological and pathogenic mechanisms underlying OCD and to personalize treatment approaches. OCD patients differ markedly from one another in both the number and the kind of comorbid disorders that they have. Large epidemiological studies have shown that, in OCD, comorbidity is the rule rather than the exception and comorbidity rates in OCD are generally higher than what would be expected based on the base rates of comorbid disorders in the general population (Miguel et al., 2008; Pinto, Mancebo, Eisen, Pagano, & Rasmussen, 2006). This suggests that, rather than a coincidental co‐occurrence of two or more independent disorders, comorbidity in OCD is a reflection of certain mechanisms linking OCD to the other disorders. It could, for instance, be that the presence of OCD instigates the development of another disorder or, conversely, that OCD and the comorbid disorder share certain risk factors, or that they are part of a larger spectrum of related disorders with overlap in etiological and pathophysiological factors. Patients with similar comorbidity profiles might share important characteristics related to etiology/pathophysiology and course of illness. Grouping patients based on their comorbidity profiles might, therefore, be a valuable way to reduce clinical heterogeneity in OCD.

The most straightforward way to group patients based on comorbidity is to focus on one comorbid disorder at a time and to investigate differences between the group of patients with the comorbid disorder and the group of patients without it. Studies of this kind have led, for instance, to the conclusion that patients with a lifetime comorbid tic disorder significantly differ from patients without it on a number of validators, warranting the inclusion in DSM‐5 of an official distinction between tic‐related and nontic‐related OCD (Leckman et al., 2010). The downside of this approach is that patients often have multiple comorbid disorders. An alternative approach is to focus on the number of disorders, by studying differences between patients with and without comorbidity or between patients with a lower versus a higher number of comorbid disorders. The problem with this approach is that adding up disorders ignores qualitative differences between comorbid disorders. Ideally, an attempt at subclassifying OCD based on comorbidity would include both the number and kind of comorbid disorders. Reasoning along these lines, Nestadt et al. (2011) identified and characterized comorbidity‐based subgroups in a sample of 706 OCD patients with a positive family history of OCD, taking into account eight comorbid disorders. They identified three subgroups: an “OCD simplex” class, with lesser comorbidity, predominantly characterized by the presence of major depressive disorder; an “OCD comorbid tic‐related” class, characterized by the predominance of tic disorder; and an “OCD comorbid affective‐related class”, with a high prevalence of panic disorder and affective syndromes. The authors found that class membership was differentially associated with other clinical characteristics.

### 1.1 Aims of the study

The present study was designed to expand on the work of Nestadt et al. by (a) using a nonfamilial sample that might be more representative of the patient population at a general OCD outpatient clinic; (b) including a broader range of comorbid disorders (i.e., 15); and (c) including longitudinal data on course of illness. The aims of the present study are as follows: (a) to determine whether subgroups of patients with OCD can be identified based on their comorbidity profiles, taking into account both number and kind of comorbidity; (b) if present, to investigate differences in clinical characteristics between these subgroups; and (c) to investigate differences in course of illness between these subgroups.

## 2 MATERIAL AND METHODS

### 2.1 Design and participants

The present study is embedded within the Netherlands Obsessive Compulsive Disorder Association (NOCDA) study, a multicenter naturalistic cohort study designed to investigate the long‐term course and outcome in OCD. Study design and baseline characteristics of the sample are described in detail elsewhere (Schuurmans et al., 2012). In short, a total number of 419 subjects aged 18 years and over with a lifetime diagnosis of OCD, as determined by the administration of the Structured Clinical Interview for DSM‐IV Axis I Disorders (SCID‐I) (First, Spitzer, Gibbon, & Williams, 1999), were included. Baseline measurements took place between 2005 and 2009. All included participants were contacted after two years for follow‐up, irrespective of their treatment status. Of the 419 participants at baseline, 311 were willing to participate in the 2‐year follow‐up assessment. During the follow‐up period, participants received treatment as usual, based on Dutch multidisciplinary guidelines. The study was accredited by the Medical Ethical committee of the Amsterdam UMC, Vrije Universiteit Amsterdam.

### 2.2 Assessment of comorbidity and covariates

#### 2.2.1 Comorbidity

The lifetime presence (yes/no) of 12 comorbid disorders (substance dependence, schizophrenia and other psychotic disorders, major depressive disorder, dysthymia, bipolar disorder, panic disorder and/or agoraphobia, specific phobia, social phobia, post‐traumatic stress disorder, generalized anxiety disorder, somatoform disorder, and eating disorder) was assessed using the Dutch version of the SCID‐I (First et al., 1999). For tic disorder, attention deficit and/or hyperactivity disorder (ADHD) and autism, a proxy diagnosis was derived from the Yale Global Tic Severity Scale (YGTSS)) (Leckman et al., 1989), the ADHD rating scale‐IV (DuPaul, Ervin, Hook, & McGoey, 1998), and the Autism‐Spectrum Quotient (AQ) (Baron‐Cohen, Wheelwright, Skinner, Martin, & Clubley, 2001), respectively. The total number of comorbid disorders investigated was thus 15.

#### 2.2.2 Covariates at baseline

Besides gender, the following clinical covariates were assessed to characterize the subgroups. A diagnosis of OCD in first‐degree relatives indicated a positive family history of OCD (yes/no) and was established with a family tree. The number of different categories of childhood trauma was inventoried with the Structured Trauma Interview (STI) (Draijer & Langeland, 1999). The age at onset for OCD was established with the SCID‐I (First et al., 1999). The severity of obsessive–compulsive symptoms was measured with the Y‐BOCS severity scale (Range 0–40) (Goodman, Price, & Rasmussen, 1989). A self‐report version of the Yale‐Brown Obsessive–Compulsive Severity scale checklist (Y‐BOCS‐SC) was used to establish the number of OCD symptoms on four dimensions: aggression/checking (20 items), symmetry/ordering (10 items), contamination/washing (9 items), and hoarding (2 items) (Goodman et al., 1989). Personality characteristics according to the Big Five (extraversion, agreeableness, conscientiousness, emotional stability, openness) were established with the 100‐item Five‐Factor Personality Inventory (Hendriks, Hofstee, & De Raad, 1999).

#### 2.2.3 Covariates at follow‐up

The presence of OCD at follow‐up was assessed with the SCID‐I (First et al., 1999). A chronic course of illness was defined as the continuous presence of at least moderately severe obsessive–compulsive symptoms in the two years between baseline and follow‐up and was retrospectively assessed at follow‐up with a Life‐Chart Interview, developed for OCD after Lyketsos, Nestadt, Cwi, Heithoff, and Eaton (1994).

### 2.3 Statistical analyses

#### 2.3.1 Identification of comorbidity‐based subgroups

To identify comorbidity‐based subgroups in the study population, we statistically modeled the occurrence of comorbidity in OCD as the expression of one or more latent classes. Dichotomous data on the presence or absence of the 15 comorbid diagnoses served as input for the latent class analysis, which was performed using MPlus v7.3 (Muthén & Muthén, 2012). For the 12 comorbid disorders assessed with the SCID‐1, data were available for all 419 subjects. For autism, ADHD, and tic disorder, data were missing for 18, 4, and 5 subjects, respectively. Using the maximum‐likelihood estimator, we followed a forward modeling approach, starting with a one‐class solution, adding one class at a time to assess improvement in model fit according to four fit indices: the Bayesian information criterion (BIC), the Akaike information criterion (AIC), the bootstrapped likelihood ratio test (BLRT), and entropy. We selected the optimal model for further analysis based on these four indices and on a substantive interpretation of the results (Van de Schoot, Sijbrandij, Winter, Depaoli, & Vermunt, 2016). The prevalence of each class and the conditional probabilities for each disorder per class were estimated, as well as each subject's posterior probability of belonging to each class. Subjects were assigned to one class based on their most likely class membership, and these data were then exported to SPSS v22 for subsequent analysis.

#### 2.3.2 Characterization of the comorbidity‐based subgroups

To further characterize the identified subgroups, we established the relations between the empirically derived class membership and the clinical covariates by performing multivariate multinomial logistic regression analyses with class membership as the dependent variable and the covariates as independent variables. To assess statistical significance for these analyses, we used a *p*‐value of <.01, in order to reduce the chance of false‐positive findings due to multiple comparisons.

#### 2.3.3 Course of illness

To assess differences in distal outcomes between the different subgroups, we compared the presence of OCD at two‐year follow‐up and illness chronicity between baseline and two‐year follow‐up between subgroups using chi‐square tests. Here, we considered a *p*‐value of <.05 to be significant.

## 3 RESULTS

### 3.1 Study sample characteristics

Baseline characteristics of the 419 subjects are presented in Table 1. The sample included 234 females (55.8%), subjects had a mean age of 36.6 years (*SD* = 10.9, range 17–79) and a mean educational level of 12.6 years (*SD* = 3.3 years). About half of the participants (52.5%) were employed and 62.2% had a partner. The mean score on the Y‐BOCS was 19.9 (*SD* = 8.1, range 0–40) reflecting a moderate mean severity of OCD. The mean age at onset of OCD was 18.5 years (*SD* = 9.6, range 4–59). 91.2% of the subjects had a current diagnosis of OCD. Major depressive disorder was by far the most prevalent comorbid disorder (56.6%), followed by tic disorder (27.3%), social phobia (23.2%), panic disorder and/or agoraphobia (22.4%) and ADHD (21.7%).

**TABLE 1:** Baseline characteristics of the total study sample (*n* = 419)

	Mean/Percentage	*SD*
Demographics		
Age	36.6	10.9
Female sex, yes	55.8%	
Education, years	12.6	3.3
Employed, yes	52.5%	
Partner, yes	62.2%	
OCD‐related		
Current OCD, yes	91.2%	
Familial, yes	41.0%	
Age at Onset, years	18.5	9.6
Y‐BOCS, total score	19.9	8.1
Comorbidity		
Major depressive disorder	237 (56.6%)	
Tic disorder	113 (27.3%)	
Social phobia	97 (23.2%)	
Panic disorder and/or agoraphobia	94 (22.4%)	
Attention deficit hyperactivity disorder	90 (21.7%)	
Substance dependence	53 (12.6%)	
Eating disorder	44 (10.5%)	
Specific phobia	43 (10.3%)	
Generalized anxiety disorder	38 (9.1%)	
Autism	27 (6.7%)	
Somatoform disorder	22 (5.3%)	
Dysthymia	22 (5.3%)	
Post‐traumatic stress disorder	19 (4.5%)	
Schizophrenia and other psychotic disorders	18 (4.3%)	
Bipolar disorder	13 (3.1%)	

Abbreviations: OCD: obsessive–compulsive disorder; *SD*: standard deviation; Y‐BOCS: Yale‐Brown Obsessive‐Compulsive Severity scale.

### 3.2 Identification of comorbidity‐based subgroups

An overview of the fit indices, class proportions, and class structure for the various models is presented in Table S1. The two‐class model outperformed the one‐class model on all four parameters, indicating that latent classes can indeed be identified in the dataset. Adding a third class improved the model fit according to the AIC, BLRT, and entropy, but not the BIC. Adding a fourth class led to further improvement in fit according to the AIC, BLRT, and entropy. Adding a fifth class led to a further marginal increase in entropy and a marginal decrease in the AIC. The six‐class model did not reach convergence. To conclude, the combination of fit indices points to a range of possible solutions with a minimum of two classes and a maximum of five classes. The final column in Table S1 shows how the two‐class model and the five‐class model are related with regard to class structure. The first two classes of the five‐class model are subgroups of the first class of the two‐class model, with overlap in class membership of 96% and 88%, respectively. The third, fourth, and fifth class of the five‐class model are subgroups of the second class of the two‐class model, with overlap in class membership of 96%, 96%, and 100%, respectively. Because the five‐class model is nested within the two‐class model and the five‐class model is more informative from a clinical perspective (see below), we will discuss both the two‐class model and the five‐class model.

**FIGURE 1:**
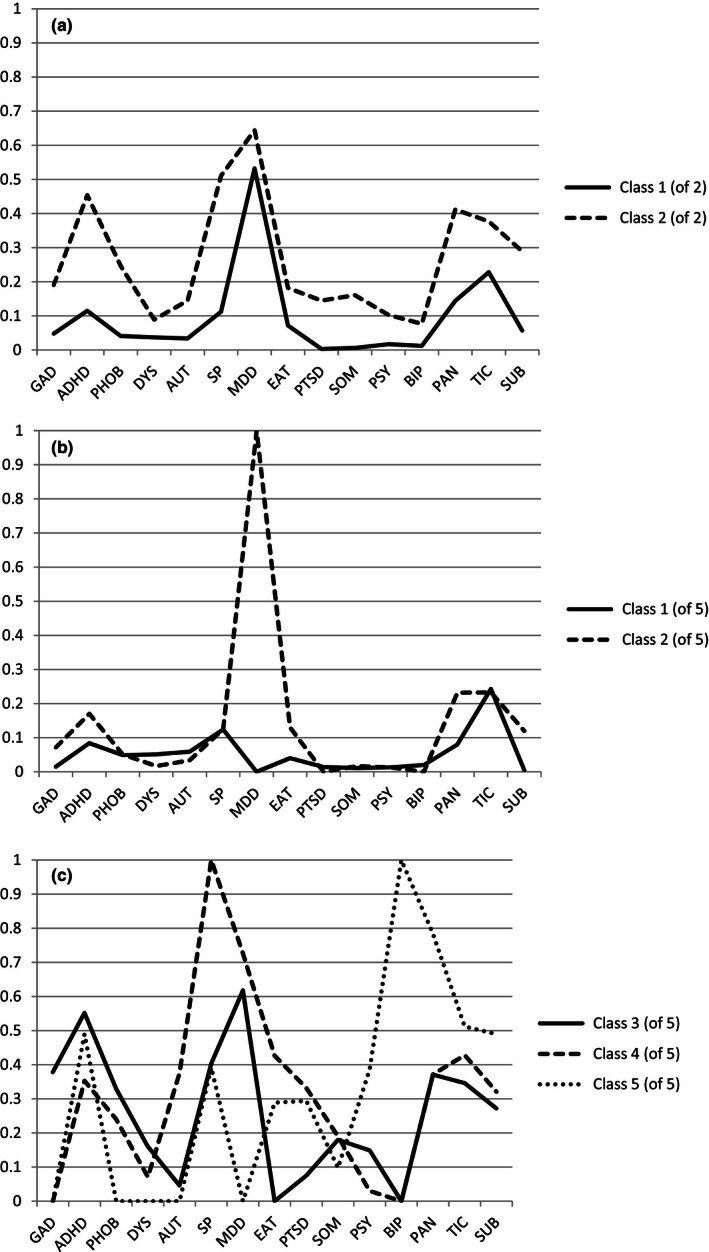
(a) Probability distributions of fifteen comorbid diagnoses for the low‐ and high‐comorbid classes (two‐class model). (b) Probability distributions of fifteen comorbid diagnoses for the two low‐comorbid classes (five‐class model). (c) Probability distributions of fifteen comorbid diagnoses for the three high‐comorbid classes (five‐class model). ADHD, attention deficit and/or hyperactivity disorder; AUT, autism; BIP, bipolar disorder; DYS, dysthymia; EAT, eating disorder; GAD, generalized anxiety disorder; MDD, major depressive disorder; PAN, panic disorder and/or agoraphobia; PHOB, specific phobia; PSY, schizophrenia and other psychotic disorders; PTSD, post‐traumatic stress disorder; SOM, somatoform disorder; SP, social phobia; SUB, substance dependence; TIC, tic disorder

The mean number of comorbid disorders and the probability distributions of the fifteen comorbid disorders for the two‐class model are presented in the top half of Table S2. To facilitate interpretation, the probability distributions are also graphically represented in Figure 1a. Class 1 of 2 contains 74% of the subjects and is characterized by a low average number of comorbid disorders (1.4). Class 2 of 2 contains 26% of the subjects and is characterized by a high average number of comorbid disorders (4.5). All comorbid disorders are more prevalent in class 2 of 2 than in class 1 of 2.

The mean number of comorbid disorders and the probability distributions of the fifteen comorbid disorders for the five‐class model are presented in the bottom half of Table S2. The first two classes of the five‐class model originate from the same superordinate low‐comorbid class and their probability distributions are graphically represented in Figure 1b. Class 1 of 5 contains 35% of the subjects and is characterized by a low prevalence of all comorbid disorders, tic disorder being the most prevalent. Notably, none of the subjects in this class have a lifetime diagnosis of MDD. Class 2 of 5 contains 44% of the subjects and is distinguished from class 1 of 5 in that all subjects in this class have a lifetime diagnosis of MDD. The probabilities of all other comorbid disorders are comparable to class 1 of 5. The three remaining classes of the five‐class model all originate from the same superordinate high‐comorbid class. They can be distinguished based on differences in comorbidity probabilities, graphically represented in Figure 1c. Class 3 of 5 contains 12% of the subjects and is the only class with a high probability of GAD. Class 4 of 5 contains 7% of the subjects and is the only class with a high probability of autism. All subjects in this class also have a lifetime diagnosis of social phobia. Class 5 of 5 is the smallest class, containing 2% of the subjects. All subjects in this class have a lifetime diagnosis of bipolar disorder. Furthermore, this class is the only one with a high probability of psychosis and the subjects in this class also have a high probability of panic disorder. The subjects without a current diagnosis of OCD (*n* = 37) were almost exclusively members of the two low‐comorbid classes (class 1 of 5: *n* = 16; class 2 of 5: *n* = 20), and only one subject was a member of the high‐comorbid class 4 of 5.

### 3.3 Clinical characteristics of the comorbidity‐based subgroups

Associations between comorbidity‐based subgroups and clinical characteristics as estimated from the multivariate multinomial logistic regression analyses are shown in Table 2. Three separate regression analyses were performed: for the two classes of the two‐class model, for the two low‐comorbid classes of the five‐class model, and for the three high‐comorbid classes of the five‐class model. The three analyses were subsequently repeated, substituting the scores on four symptom dimensions by the scores on five personality domains (results described in text below, not shown in Table 2), as sparse data prevented combining these covariates in one model.

**TABLE 2:** Clinical characteristics of the comorbidity‐based classes at baseline

Two‐class model	Class 1	Class 2	2 versus 1
Class label	Low comorbid	High comorbid	
Class size	311 (74%)	108 (26%)	
Mean no. of comorbid disorders (*SD*)	1.4 (1.0)	4.5 (1.3)	
			**OR**
Female sex, yes (%)	55.9	55.6	0.75 (0.44–1.30)
Familial, yes (%)	39.2	46.3	1.06 (0.62–1.81)
Childhood trauma	1.3	1.9	1.48 (1.18–1.86)*
Age at onset	19.2	16.5	0.98 (0.95–1.02)
Illness Severity	18.8	23.1	1.09 (1.05–1.13)**
Symptom dimensions			
Aggression/Checking	6.1	8.4	1.11 (1.03–1.18)*
Symmetry/Ordering	3.7	4.5	0.98 (0.90–1.08)
Contamination/Washing	2.9	3.4	0.92 (0.82–1.02)
Hoarding	0.4	0.5	1.20 (0.83–1.73)

Five‐class model	Class 1	Class 2	2 versus 1	Class 3	Class 4	Class 5	4 versus 3	5 versus 4	5 versus 3
Class label	Simplex	MDD		GAD	AUT/SP	PSY/BIP			
Class size	147 (35%)	186 (44%)		49 (12%)	27 (7%)	10 (2%)			
Mean no. of comorbid disorders (*SD*)	0.8 (0.9)	2.2 (0.9)		4.4 (1.1)	5.1 (1.6)	4.8 (1.3)			
			**OR**				**OR**	**OR**	**OR**
Female sex, yes (%)	50.3	58.6	1.15 (0.71–1.88)	46.9	77.8	70.0	2.72 (0.64–11.54)	0.68 (0.10–4.64)	1.84 (0.30–11.37)
Familial, yes (%)	34.2	42.2	1.22 (0.74–2.00)	42.9	51.9	80.0	2.49 (0.63–9.76)	1.70 (0.22–13.40)	4.23 (0.59–30.43)
Childhood trauma	1.2	1.4	1.14 (0.90–1.44)	1.7	2.3	2.8	1.32 (0.82–2.11)	1.03 (0.59–1.82)	1.36 (0.75–2.46)
Age at Onset	19.4	18.4	1.00 (0.98–1.03)	19.0	15.8	11.3	1.03 (0.95–1.12)	0.88 (0.76–1.01)	0.91 (0.79–1.04)
Illness Severity	18.2	19.9	1.02 (0.99–1.06)	21.3	26.2	20.9	1.08 (0.98–1.19)	0.86 (0.74–1.00)	0.93 (0.80–1.08)
Symptom dimensions									
Aggression/Checking	5.5	6.6	1.07 (1.00–1.15)	8.8	8.8	10.2	0.82 (0.68–0.98)	1.12 (0.88–1.43)	0.92 (0.73–1.16)
Symmetry/Ordering	3.3	3.9	1.03 (0.94–1.12)	4.2	5.9	6.4	1.44 (1.08–1.93)^t^	0.99 (0.67–1.45)	1.42 (0.96–2.12)
Contamination/Washing	2.6	3.2	1.03 (0.93–1.14)	3.5	3.4	3.2	0.84 (0.65–1.09)	1.10 (0.73–1.65)	0.92 (0.62–1.37)
Hoarding	0.4	0.4	0.84 (0.58–1.22)	0.5	0.6	0.6	0.88 (0.40–1.94)	0.92 (0.29–2.92)	0.81 (0.26–2.47)

Abbreviations: AUT, autism; BIP, bipolar disorder; GAD, generalized anxiety disorder; MDD, major depressive disorder; OR, odds ratio; PSY, schizophrenia and other psychotic disorders; *SD*, standard deviation; SP, social phobia.

^t^ *p*(trend) = .013.

* *p* < .01.

** *p* < .001.

Compared to the low‐comorbid class (class 1 of 2), membership of the high‐comorbid class (class 2 of 2) was associated with a higher childhood trauma score (OR = 1.48 (1.18–1.86), *p* < .01), a higher illness severity (OR = 1.09 (1.05–1.13), *p* < .001), a higher score on the aggression/checking symptom dimension (OR = 1.11 (1.03–1.18), *p* < .01) and lower scores on extraversion (OR = 0.68 (0.54–0.86), *p* < .01), conscientiousness (OR = 0.67 (0.52–0.87), *p* < .01), and emotional stability (OR = 0.68 (0.53–0.88), *p* < .01). Compared to the low‐comorbid simplex class (class 1 of 5), membership of the low‐comorbid MDD class (class 2 of 5) was associated with a lower score on emotional stability (OR = 0.66 (0.51–0.85), *p* < .01). Compared to the high‐comorbid GAD class (class 3 of 5), membership of the high‐comorbid autism/social phobia class (class 4 of 5) was associated with a higher score on the symmetry/ordering symptom dimension (OR = 1.44 (1.08–1.93), *p*(trend) = .013). There were no significant differences in clinical characteristics between the high‐comorbid psychosis/bipolar class (class 5 of 5) and the two other high‐comorbid classes.

### 3.4 Course of illness

The rate of remission of OCD after two years was lower in the high‐comorbid class (class 2 of 2) compared to the low‐comorbid class (class 1 of 2), although the difference was not significant (χ^2^) = 3.5, *p* = .06) (see Table S3). Moreover, subjects in the high‐comorbid class had a significantly higher chance of having a chronic course of illness between baseline and follow‐up compared to subjects in the low‐comorbid class (χ^2^ = 4.5, *p* = .03). Rates of remission and course of illness did not differ significantly between the two low‐comorbid classes (class 1 of 5 and class 2 of 5) or between the three high‐comorbid classes (class 3 of 5, class 4 of 5, and class 5 of 5).

## 4 DISCUSSION

The two‐class model discerns a larger low‐comorbid class from a smaller high‐comorbid class. As all comorbid disorders are more prevalent in the high‐comorbid class than in the low‐comorbid class, the two classes can be construed as lying on a dimensional scale of general comorbidity burden. Given the purely quantitative nature of the distinction between the two classes, the clinical characteristics associated with membership of the high‐comorbid class are best considered risk factors for psychopathology in general rather than truly distinct subgroups of OCD patients. We will therefore focus our interpretation of the results on the more fine‐grained level of the five‐class model. If only the average amount of comorbid disorders is taken into account (see Table 2), the five classes could also be construed as lying on a dimensional scale of general comorbidity burden, similar to the two‐class model. From a purely quantitative perspective, the low‐comorbid simplex class (class 1 of 5) would be followed by an intermediate comorbid MDD‐related class (class 2 of 5) and then followed by the combined three high‐comorbid classes (classes 3, 4, and 5 of 5). However, the probability distributions as shown in Figure 1b make it clear that the MDD‐related class is not just an intermediate class, but uniquely characterized by a 100% prevalence of MDD and a low prevalence of all other disorders. Similarly, as shown in Figure 1c, the three high‐comorbid classes, although roughly equal from a quantitative perspective, significantly differ from each other in their comorbidity profile. The five‐class structure is thus based on both quantitative and qualitative differences between the classes.

The low‐comorbid simplex class (class 1 of 5) contained all 64 subjects with no lifetime comorbidity. Torres et al. (2013) investigated the characteristics of OCD patients without lifetime comorbidity (“pure OCD”) and found that these patients were more likely to be female, to have lower scores on depression, anxiety, and suicidality scales and were less likely to have had psychotherapy. The fact that our simplex class also contained a significant amount of subjects with a low amount of non‐MDD comorbidity makes it difficult to directly compare the groups. However, the results presented in Table 2 clearly show that, in terms of childhood trauma burden, age at onset, general and symptom‐specific illness severity and course of illness, this class has the most favorable clinical profile.

In the low‐comorbid range, the presence of lifetime MDD comorbidity clearly distinguished class 2 from class 1. The association between a lower score on emotional stability, that is, higher neuroticism, and membership of the MDD‐related class follows previous research that has identified neuroticism as the key personality trait contributing to comorbidity in the spectrum of affective and anxiety disorders (Bienvenu et al., 2004; Kampman, Viikki, & Leinonen, 2017; Rector, Bagby, Huta, & Ayearst, 2012; Spinhoven, Rooij, Heiser, Smit, & Penninx, 2009). The fact that MDD is also highly prevalent in the two larger high‐comorbid classes (see Figure 1c) suggests that MDD is not a defining disorder in the overall class structure but rather a common accompaniment of OCD in general. Indeed, MDD is consistently found to be the most prevalent comorbid disorder in OCD (Pinto et al., 2006; Torres et al., 2016) and most often occurs after the onset of OCD (Demal, Lenz, Mayrhofer, Zapotoczky, & Zitterl, 1993). The high occurrence of comorbid MDD is often explained as the result of chronic distress and impairment in psychosocial functioning associated with OCD (Quarantini et al., 2011). Other potential mechanisms linking the two disorders include OCD‐specific cognitive distortions (Abramowitz, Storch, Keeley, & Cordell, 2007), direct symptom–symptom interactions (McNally, Mair, Mugno, & Riemann, 2017), shared underlying emotional vulnerabilities (Chasson, Bello, Luxon, Graham, & Leventhal, 2017), and shared genetic vulnerabilities (Bolhuis et al., 2014).

In the high‐comorbid range, three classes could be distinguished, all with roughly the same average number of comorbid disorders but with several interesting differences in comorbidity profiles. The smallest of these classes is class 5, which is uniquely characterized by high loadings of psychotic disorder, bipolar disorder, and panic disorder and an absence of MDD. The separation of this class from all other classes has strong statistical support, as the emergence of this class in the three‐class model is accompanied by a significant decrease in the AIC and a significant increase in entropy (Table S1). The association between OCD and psychotic disorders has been given considerable attention in previous research. First of all, there can be symptomatic overlap between the two in the case of OCD with poor or absent insight, where the beliefs underlying the obsessive–compulsive symptoms have become delusional. Distinguishing between this type of OCD and psychotic disorder is a matter of differential diagnosis. Additionally, a fair amount of OCD patients also fulfill the full criteria for a comorbid psychotic disorder, with estimates ranging between 2.7% and 4.7% (Pinto et al., 2006; Schuurmans et al., 2012). This comorbidity rate been explained by shared etiological (genetic) factors (Swets et al., 2015) and the OCD‐inducing effect of certain atypical antipsychotic drugs, possibly explained by their impact on serotonergic neurotransmission in brain areas associated with OCD (Fonseka, Richter, & Müller, 2014). The prevalence of OCD is also relatively high in patients with bipolar disorder. Studies on possible associations suggest that OCD often occurs after the onset of bipolar disorder, with obsessive–compulsive symptoms typically worsening during depressive episodes and improving during manic episodes (Amerio et al., 2015; Perugi et al., 2002). The fact that panic disorder and/or agoraphobia was also prevalent (albeit not exclusively) in this particular subgroup is in line with previous research that suggests that the presence of panic disorder with agoraphobia was predictive of bipolar disorder comorbidity in adult patients with OCD (Domingues‐Castro, Torresan, & Shavitt, 2019). In this context, it is important to note that all subjects included in our sample were actively seeking treatment and were referred to one of the contributing mental health centers with OCD as their primary (suspected) diagnosis. This may have led to an underrepresentation of OCD patients with comorbid psychotic and/or bipolar disorders, as these patients are generally more prone to avoid seeking treatment and, when they do seek treatment, are usually treated in different echelons of the Dutch mental health care system. This might explain the relatively low prevalence of comorbid psychotic and bipolar disorders in our sample and the small size of class 5 that groups these subjects together; this, consequently, makes it difficult from a statistical perspective to properly characterize this important subgroup in terms of clinical features. The high percentage of a positive family history of OCD and the early age of onset could point to a distinct phenotype, but more studies with enriched samples are needed to investigate this further.

The two remaining classes in the high‐comorbid range are characterized by a high loading of GAD (class 3) and high loadings of autism and social phobia (class 4), respectively. Importantly, the statistical support for the distinction between these two classes is limited, as their separation in the five‐class model was only accompanied by a marginal decrease in AIC and a marginal increase in entropy (see Table S1). Notwithstanding this fact, the qualitative differences between these classes are interesting to discuss in the light of two other nosological distinctions within the domain of affective and anxiety disorders that have been investigated in recent years. The first distinction originates from attempts to explain comorbidity patterns among affective and anxiety disorders using factor analysis. A consistent finding was the distinction between a so‐called “anxious‐misery” factor that included major depressive disorder, dysthymia, and generalized anxiety disorder, and a “fear” factor that included panic disorder and the phobic disorders (Krueger, 1999; Vollebergh et al., 2001). The predominance of GAD in class 3 and the high prevalence of social phobia in class 4 show some similarity to this distinction, although it must be said that the other disorders involved are evenly distributed between the classes. The second distinction is related to the spectrum of obsessive–compulsive and related disorders (OCRDs) that became a separate nosological category with the introduction of the DSM‐5 (Phillips et al., 2010). In the developmental stages of the DSM‐5, arguments were put forward to distinguish within the obsessive–compulsive spectrum a group of disorders characterized by so‐called “lower order” repetitive and/or stereotypic behaviors and a group of disorders characterized by so‐called “higher order” compulsive behaviors primarily aimed at anxiety reduction (Phillips et al., 2010). The high prevalence of GAD in class 3 relative to class 4 suggests that it resembles (in a way) the group of higher order OCRDs. Likewise, the high prevalence of autism in class 4 and the predominance of obsessions of symmetry and ordering compulsions that appear to be aimed more at relieving “not just right feelings” rather than being aimed at pure anxiety reduction (Coles, Frost, Heimberg, & Rhéaume, 2003; Pietrefesa & Coles, 2008), suggest that this class resembles (in a way) the group of lower‐order OCRDs. The high prevalence of both autism and social phobia in class 4 could at least in part be due to phenomenological overlap between the criteria for both disorders. Interestingly, the presence of tic disorder, previously associated with autism comorbidity in OCD (Anholt et al., 2010) and with symmetry/ordering compulsions (Huisman‐van Dijk, Schoot, Rijkeboer, Mathews, & Cath, 2016), did not discriminate between the two classes.

### 4.1 Previous research

Several notable differences in study design make it difficult to directly compare our results to those of Nestadt et al. (2011). In their study, the sample size was larger (*n* = 706), all subjects had a positive family history of OCD and an early age at onset of OCD and the sample included both children and adults. Only eight comorbid disorders were taken into account: generalized anxiety disorder, major depressive disorder, panic disorder, separation anxiety disorder, tic disorder, mania, somatization disorders, and grooming disorders. Additionally, Nestadt et al. used both “definite diagnoses” and “probable diagnoses” (most but not all DSM‐criteria met) to boost comorbidity rates. Despite these significant differences in design, the three‐class solution that they found, all containing one‐third of the subjects, was fairly comparable to our five‐class solution. The first class that Nestadt et al. distinguished was characterized by a low average number of comorbid disorders, comparable to our class 1 of 5. The second class they distinguished was characterized by an intermediate number of comorbid disorders, comparable to our class 2 of 5, except that MDD was less prevalent and GAD and tic disorder were more prevalent in their class. Finally, the third class they distinguished was characterized by a high number of all comorbid disorders, comparable to our classes 3, 4, and 5 of 5 combined. Compared to the previous study, the main additional finding of the present study is the further subdivision of the highly comorbid class in three qualitatively distinct and potentially interesting subgroups.

### 4.2 Limitations and future directions

Strengths of this study include the extensive clinical phenotyping of the cohort, the representativeness of the sample, positively influencing the external validity of the results with regard to OCD patients treated in outpatient clinics and the relatively large sample size. The inclusion of a broad spectrum of well‐defined comorbid disorders allowed us to refine and expand on previous work. Several limitations also have to be addressed. Firstly, as mentioned above, the way the sample was selected probably resulted in an underrepresentation of subjects with comorbid psychotic disorder and/or bipolar disorder. To address this problem, future studies could benefit from enriching outpatient‐based samples with subjects from other echelons of the mental healthcare system. A second limitation of this study concerns the lack of data on several comorbid diagnoses that are considered part of the OCD spectrum, most notably body dysmorphic disorder and grooming disorders, and other diagnoses of interest such as illness anxiety disorder and impulse‐control disorders (Phillips et al., 2010). Inclusion of these disorders might have resulted in a different class structure. Finally, the small group size of the three high‐comorbid subgroups may have led to the occurrence of type‐II errors. Future studies to replicate and expand on our findings are therefore needed.

This study shows that the application of latent class analysis to a well‐characterized sample of OCD patients is a promising way to reduce clinical heterogeneity. Rather than considering comorbidity a hindrance, its presence could be embraced as a vital and valuable source of information and key to a better understanding of OCD. Grouping OCD patients based on their comorbidity profile might provide more homogeneous, and therefore more suitable, categories for future studies aimed at unraveling the etiological mechanisms underlying this debilitating disorder.

## CONFLICT OF INTEREST

None declared.

## AUTHOR CONTRIBUTIONS

LO, RS, AH, GM and AB were involved in the conception and design of the study and the analysis and interpretation of the data. LO, RS, AH, PO, MK, GM and AB drafted and revised the article. LO, RS, AH, PO, MK, GM and AB gave final approval.

## Supporting information

TableS1‐S3

## Data Availability

The data that support the findings of this study are available from the corresponding author upon reasonable request.
