# Supplementary material for: Classification of comorbidity in obsessive–compulsive disorder: A latent class analysis
Source: Brain Behav. 2020 May 13;10(7):e01641. doi: 10.1002/brb3.1641 (PMC7375063; doi:10.1002/brb3.1641)
Supplement: Supplementary file 1 — TableS1‐S3 [file BRB3-10-e01641-s001.docx]

Supplementary Table 1. Fit indices, class proportions and class structure for one- to six-class models.

| **Model** | **BIC** | **AIC** | **BLRT** | **Entropy** | **Class proportions (%)** | **Class structure^1^** |
| --- | --- | --- | --- | --- | --- | --- |
| 1 class | 4577 | 4516 | NA | 1 | 100 | 1&2&3&4&5 |
| 2 classes | **4541** | 4415 | < 0.001 | 0.59 | 74 / 26 | 1&2 / 3&4&5 |
| 3 classes | 4579 | 4389 | < 0.001 | 0.73 | 72 / 25 / 3 | 1&2 / 3&4 / 5 |
| 4 classes | 4630 | 4375 | **< 0.001** | 0.82 | 44 / 34 / 20 / 2 | 1 / 2 / 3&4 / 5 |
| 5 classes | 4693 | **4374** | 0.15 | **0.85** | 44 / 35 / 12 / 7 / 2 | 1 / 2 / 3 / 4 / 5 |
| 6 classes | 4758 | 4375 | 0.67 | 0.85 | 41 / 33 / 14 / 8 / 3 / 1 | NA |

Abbreviations: BIC: Bayesian Information Criterion; AIC: Akaike Information Criterion; BLRT: Bootstrapped Likelihood Ratio Test

Bold: optimal parameter value; NA: not available (due to convergence problems)

^1)^The class structure displays the (approximate) hierarchical structure of the classes found in the five-class model

Supplementary Table 2. Probability distributions of fifteen comorbid diagnoses for the two-class model and five-class model.

| **Two class model ^a^** | **Class 1** | | **Class 2** | | |
| --- | --- | --- | --- | --- | --- |
| **Class label** | Low comorbid | | High comorbid | | |
| **Class size** | 311 (74%) | | 108 (26%) | | |
| **Mean no. of comorbid disorders (SD)** | 1.4 (1.0) | | 4.5 (1.3) | | |
| *Comorbid disorder* | *Prob (SE)* | | *Prob (SE)* | | |
| **GAD** | 0.05 (0.02) | | **0.19** (0.05) | | |
| **ADHD** | 0.12 (0.04) | | **0.45** (0.07) | | |
| **PHOB** | 0.04 (0.02) | | **0.25** (0.05) | | |
| **DYS** | 0.04 (0.01) | | **0.09** (0.03) | | |
| **AUT** | 0.03 (0.02) | | **0.15** (0.04) | | |
| **SP** | 0.11 (0.03) | | **0.51** (0.10) | | |
| **MDD** | 0.53 (0.04) | | **0.64** (0.06) | | |
| **EAT** | 0.07 (0.02) | | **0.18** (0.05) | | |
| **PTSD** | 0.00 (0.01) | | **0.15** (0.05) | | |
| **SOM** | 0.01 (0.01) | | **0.16** (0.04) | | |
| **PSY** | 0.02 (0.02) | | **0.10** (0.04) | | |
| **BIP** | 0.01 (0.01) | | **0.08** (0.03) | | |
| **PAN** | 0.14 (0.03) | | **0.41** (0.07) | | |
| **TIC** | 0.23 (0.03) | | **0.38** (0.06) | | |
| **SUB** | 0.06 (0.02) | | **0.29** (0.06) | | |
| **Five-class model ^b^** | **Class 1** | **Class 2** | **Class 3** | **Class 4** | **Class 5** |
| **Class label** | Simplex | MDD | GAD | AUT/SP | PSY/BIP |
| **Class size** | 147 (35%) | 186 (44%) | 49 (12%) | 27 (7%) | 10 (2%) |
| **Mean no. of comorbid disorders (SD)** | 0.8 (0.9) | 2.2 (0.9) | 4.4 (1.1) | 5.1 (1.6) | 4.8 (1.3) |
| *Comorbid disorder* | *Prob (SE)* | *Prob (SE)* | *Prob (SE)* | *Prob (SE)* | *Prob (SE)* |
| **GAD** | 0.02 (0.01) | 0.07 (0.02) | **0.38** (0.17) | 0.00 (0.00) | 0.00 (0.00) |
| **ADHD** | 0.08 (0.04) | 0.17 (0.04) | **0.55** (0.12) | 0.35 (0.15) | 0.49 (0.16) |
| **PHOB** | 0.05 (0.03) | 0.05 (0.02) | **0.33** (0.13) | 0.24 (0.12) | 0.00 (0.00) |
| **DYS** | 0.05 (0.02) | 0.02 (0.01) | **0.16** (0.07) | 0.07 (0.06) | 0.00 (0.00) |
| **AUT** | 0.06 (0.03) | 0.03 (0.02) | 0.05 (0.08) | **0.37** (0.17) | 0.00 (0.00) |
| **SP** | 0.12 (0.04) | 0.13 (0.04) | 0.40 (0.12) | **1.00** (0.00) | 0.39 (0.16) |
| **MDD** | 0.00 (0.00) | **1.00** (0.00) | 0.62 (0.10) | 0.73 (0.13) | 0.00 (0.00) |
| **EAT** | 0.04 (0.02) | 0.13 (0.03) | 0.00 (0.00) | **0.43** (0.18) | 0.29 (0.14) |
| **PTSD** | 0.01 (0.01) | 0.00 (0.00) | 0.08 (0.05) | **0.33** (0.15) | 0.29 (0.15) |
| **SOM** | 0.01 (0.01) | 0.02 (0.02) | 0.18 (0.08) | **0.19** (0.12) | 0.10 (0.09) |
| **PSY** | 0.01 (0.01) | 0.01 (0.02) | 0.15 (0.07) | 0.03 (0.09) | **0.39** (0.15) |
| **BIP** | 0.02 (0.01) | 0.00 (0.00) | 0.00 (0.00) | 0.00 (0.00) | **1.00** (0.00) |
| **PAN** | 0.08 (0.03) | 0.23 (0.04) | 0.37 (0.14) | 0.37 (0.12) | **0.78** (0.15) |
| **TIC** | 0.24 (0.05) | 0.23 (0.04) | 0.35 (0.16) | 0.43 (0.16) | **0.51** (0.16) |
| **SUB** | 0.01 (0.01) | 0.12 (0.03) | 0.27 (0.09) | 0.32 (0.15) | **0.49** (0.16) |

Abbreviations: SD: standard deviation; Prob: probability; SE: standard error; GAD: generalized anxiety disorder; ADHD: attention deficit and/or hyperactivity disorder; PHOB: specific phobia; DYS: dysthymia; AUT: autism; SP: social phobia; MDD: major depressive disorder; EAT: eating disorder; PTSD: post-traumatic stress disorder; SOM: somatoform disorder; PSY: schizophrenia and other psychotic disorders; BIP: bipolar disorder; PAN: panic disorder and/or agoraphobia; TIC: tic disorder; SUB: substance dependence

Probabilities are displayed in **bold face** in the class with the highest conditional probability.

^a^ See Figure 1a

^b^ See Figure 1b and 1c

Supplementary Table 3. Clinical characteristics of the comorbidity-based classes at follow-up.

| **Two-class model** | **Class 1**  **Low comorbid** | | | **Class 2**  **High comorbid** | | | | **2 vs 1** |
| --- | --- | --- | --- | --- | --- | --- | --- | --- |
| OCD, yes (%) | 65.6 | | | 77.6 | | | | *Χ^2^*  3.5 |
| Chronic course, yes (%) | 31.9 | | | 46.2 | | | | 4.5* |
| **Five-class model** | **Class 1**  **Simplex** | **Class 2**  **MDD** | **2 vs 1** | **Class 3**  **GAD** | **Class 4**  **AUT/SP** | **Class 5**  **PSY/BIP** | **5 vs 4 vs 3** |  |
| OCD, yes (%) | 64.3 | 67.6 | *Χ^2^*  0.3 | 81.3 | 80.0 | 50.0 | *Χ^2^*  2.1 |  |
| Chronic course, yes (%) | 31.2 | 34.0 | 0.2 | 46.9 | 57.1 | 46.0 | 4.1 |  |

Abbreviations: OCD: Obsessive-Compulsive Disorder; MDD: major depressive disorder; GAD: generalized anxiety disorder; AUT: autism; SP: social phobia; PSY: schizophrenia and other psychotic disorders; BIP: bipolar disorder
